# Barriers in the School-Based Pan-Gender HPV Vaccination Program in Sweden: Healthcare Providers’ Perspective

**DOI:** 10.3390/vaccines11020310

**Published:** 2023-01-30

**Authors:** Ida Enskär, Karin Enskär, Tryggve Nevéus, Andrea Hess Engström, Maria Grandahl

**Affiliations:** Department of Women’s and Children’s Health, Uppsala University, 751 85 Uppsala, Sweden

**Keywords:** barriers, children, head and neck cancer, human papillomavirus, HPV vaccination, healthcare providers, immunization program, school health, school nurses

## Abstract

Background: Human papillomavirus (HPV) vaccines effectively prevent, and can even eliminate, HPV-related cancers. Currently, vaccination rates are suboptimal in the national Swedish school-based vaccination program. School nurses play a key role in all aspects of the vaccination process. Therefore, this study aims to explore school nurses’ perceived HPV vaccination challenges. Methods: Seven focus group interviews were conducted with school nurses (*n* = 35) working in nine socio-demographically diverse municipalities in mid-Sweden. Data were analyzed using qualitative content analysis. Results: Participants described difficulties in encountering and handling the diversity of reasons for vaccine hesitancy. Parents known to be skeptical of vaccines in general were seen as most difficult to reach. Uncertainty was expressed concerning the extent of professional responsibility for vaccine promotion. The informants expressed a lack of guidelines for vaccine promotion and described challenges in supporting the child’s own wishes. Creating a safe space for the individual child was seen as crucial. Other problems described were the challenges of overcoming children’s fear of needles, supporting unvaccinated children, and being confronted with the remaining gender inequities of the pan-gender vaccination program. Conclusions: Our results suggest that school nurses, especially those new to their profession, may benefit from training and guidance22 material on how to address vaccine hesitancy.

## 1. Introduction

Human papillomavirus (HPV) infections are substantial contributors to the global cancer burden, predominately cervical cancer, but also cancer in the head and neck, anus, vulva, vagina, and penis [1,2]. A major increase in the incidence of HPV-positive tonsil and tongue base cancer, mostly affecting men, has been seen in Sweden and other Western countries [2,3]. HPV vaccines effectively prevent HPV-related cancers and are part of the World Health Organization’s (WHO) strategy to eliminate cervical cancer [4,5,6]. Still, vaccination rates remain suboptimal globally. Furthermore, pan-gender vaccination programs have been called for and implemented in several countries in order to achieve equal and effective prevention of HPV-related cancer [7].

In Sweden, all children are offered the HPV vaccination in fifth grade (11–12 years). The vaccine is provided by the school health services free of charge but requires parental consent. Among Swedish children born in 2009, 83% of all girls and 77% of all boys were fully vaccinated [8]. These numbers are substantially lower compared to other childhood vaccinations in the Swedish vaccination program, which have a coverage of >97%. Furthermore, in several municipalities vaccine coverage is far below the national average [8].

Vaccine hesitancy, i.e., being reluctant to, delaying, or declining recommended vaccinations, is a growing problem [9,10]. In 2019, the WHO classified this as one of the top ten global public health threats [11]. Vaccine hesitancy is a complex, context-specific, and multi-factorial problem that varies both over time and between different vaccines and needs to be addressed continuously [12,13].

HPV vaccination has since its introduction faced barriers due to the spread of misinformation and controversy regarding its prevention of sexually transmitted infections (STIs) [14]. Furthermore, previous research shows that parents’ and adolescents’ knowledge about HPV and the vaccine is generally low. Concerns about vaccine safety, insufficient information, as well as the young age of the child, are reported reasons for parents not consenting to the vaccination. Other barriers include a low perceived risk of getting an HPV infection or HPV-related cancer. Lower socioeconomic status correlates to lower uptake. Finally, cultural norms, moral values, and religious beliefs also influence parental decisions [15,16,17,18].

Healthcare providers (HCPs) constitute one of the most important resources of vaccine information, and parents’ trust in their recommendations is crucial for the acceptance of childhood vaccinations [14,19]. HCPs’ knowledge, attitudes, and communication skills are important aspects for effective vaccine recommendations [20,21]. They face several challenges. Parental vaccine hesitancy or wish to postpone the vaccination, as well as lack of time to provide adequate information, are common barriers in many countries. Furthermore, the misconceptions that HPV is a female problem and that vaccination can lead to promiscuity can influence the decision [22]. HCPs request more information about HPV in order to meet parents and children’s questions and concerns [23,24,25]. Other expressed barriers include the discomfort when discussing the link between HPV and sexual behavior, especially when explaining oral and anal HPV transmission [26].

In Sweden, school nurses are responsible for all aspects of HPV vaccination and therefore have a prominent role in the success of the national vaccination program. A previous study has shown that Swedish school nurses have favorable attitudes toward HPV vaccinations and the introduction of the pan-gender vaccination program. However, the study revealed a need for more HPV education and training in the provision of information about HPV to parents and children [27].

Further efforts should be made to improve suboptimal vaccination rates. The Immunization Agenda 2030 states that no child should be left behind [28]. To achieve this goal, i.e., promote children’s health and prevent future HPV-related cancer, we need a deeper understanding of HCPs’ perceived challenges for vaccine acceptance. To our knowledge, no such studies have been conducted since the introduction of the pan-gender vaccination program in 2020 in Sweden. Therefore, this study aimed to explore school nurses’ perceived challenges to the pan-gender HPV vaccination program.

## 2. Materials and Methods

This explorative interview study was conducted as one of three qualitative studies involving key stakeholders in HPV vaccination: school nurses, parents, and children. The study is presented in accordance with Standards for Reporting Qualitative Research to improve transparency and is reported according to COREQ-checklist [29,30]. Following the Medical Research Council’s guidelines, the results are used in the development phase of an intervention aimed to increase vaccination rates in Swedish municipalities with low vaccine coverage [31].

### 2.1. Design

Focus group (FG) interviews with school nurses were conducted as a means for participants to share their perceptions and, by facilitating discussion, shed light on both common and differing experiences [32].

### 2.2. Setting and Participants

In Sweden, HPV vaccination was implemented in the school-based vaccination program for girls in 2012 and included catch-up vaccinations for girls up to the age of 18. The pan-gender vaccination program was introduced in 2020, but no catch-up vaccinations are offered to older boys (born before 2009). Sweden is divided into 21 regions and 290 municipalities. The regions are responsible for healthcare services and the municipalities are responsible for school health services including the provision of vaccinations included in the childhood vaccination program to children 6–18 years of age. In an effort to recruit school nurses experienced in the subject of interest, municipalities with lower HPV vaccine coverage, according to available statistics by the public health agency, were approached. In total, 13 diverse municipalities in north-mid Sweden were contacted. In each municipality, the head of school health services approved and facilitated contact with school nurses eligible for the study. Furthermore, they advised on socio-demographic differences between schools as well as recent vaccine-coverage statistics.

A purposive sampling method was used to recruit school nurses working in urban and rural areas, in public and private schools as well as in socio-economically different catchment areas. School nurses working in middle schools (grades 1–6) who had at least one semester’s experience in offering HPV vaccinations were eligible to participate. Participants were contacted and received an information letter directly from the head of school health services or the researchers. Furthermore, snowball sampling was used. Participants gave oral and written consent and completed a survey of background questions.

Information power in qualitative research can be evaluated by richness of data and high specificity experiences of the phenomenon among participants [33]. Information power was continuously discussed and evaluated by the research group to decide upon the number of FGs needed.

### 2.3. Data Collection

Interviews were conducted between November 2021 and April 2022. The time and place for the interview were decided by the participants. However, due to the COVID-19 pandemic social restrictions most of the interviews were conducted digitally through Zoom (*n* = 6). All participants joined with video and participated from their preferred location, either from home or their office.

A semi-structured interview guide with three central, open-ended questions was used to explore school nurses’ experiences regarding HPV and the HPV vaccination and their own views and knowledge on the subject, as well as their experience of providing information to parents and children. Follow-up questions, focused on perceived challenges, were asked to clarify or expand the participants’ reasoning.

The interviews were moderated by the first author or MSc students, together with an observer from the research group (another PhD student or senior researcher). The role of the observer was to support the moderator, take notes, and ask follow-up questions if necessary. At the end of each interview, the observer summarized the content of the discussion so that the participants could confirm that their perceptions had been clearly understood or add to their reasoning. Interviews were audio recorded, lasted 50–90 min, and were transcribed verbatim without delay.

### 2.4. Data Analysis

Data were analyzed with an inductive approach using content analysis [34]. Initially, the transcripts were read multiple times in order for the researchers to familiarize themselves with the data and get a sense of the overall content. Meaning-bearing units related to the aim were then extracted and condensed by deleting hesitations, repetitions, and pauses. The condensed meaning-bearing units were then labeled with a descriptive code. During this process, the participant’s wording was maintained in order not to lose the core meaning of the data. Data units not directly reflecting the aim were kept as context for the meaning-bearing unit. Codes with similar content were then sorted into subcategories and finally grouped into categories.

The analysis was a back-and-forth process between the original transcripts and the different components of the analysis. The categories were formed after reaching a consensus during discussions in the research group. An example of the analysis process is presented in Table 1.

## 3. Results

In total, seven focus group interviews were conducted with school nurses (*n* = 35) working in nine different municipalities. The participants were all female and their background characteristics are presented in Table 2.

The analysis resulted in three categories describing school nurses’ perceived challenges for HPV vaccination:

1. Addressing various reasons for vaccine hesitancy;
2. Interpreting professional responsibilities;
3. Creating a safe space for the individual child.

A summary of sub-categories and categories is given in Table 3 and presented below, with illustrative quotations of the findings.

### 3.1. Addressing Various Reasons for Vaccine Hesitancy

#### 3.1.1. Encountering Hesitant Parents

School nurses experienced vaccine uptake to be generally good and perceived that HPV vaccination had become more accepted since first introduced, reflected by higher vaccination rates and fewer questions from parents. Still, participants had frequently encountered hesitant parents and parents with outspoken general vaccine skepticism. Fear of side effects was perceived to have decreased over time but was still present. Similarly, lack of knowledge of the vaccine and its inclusion in the childhood vaccination program was also experienced as a lingering reason for hesitancy, especially among parents of boys. Hesitancy towards the vaccine was described as multifaceted and very context-specific. Challenges of addressing parents’ concerns that the school nurse experienced at one school could completely differ from another school. Furthermore, school nurses experienced that the difficulty in communicating with parents varied depending on the reasons for their hesitancy.

An underlying perceived cause of parental concern specific to the HPV vaccination was its protection against a sexually transmitted infection (STI). Moral or religious reasons, in particular, were experienced as being challenging to discuss; for example, parents’ expectations that their child would only have one sexual partner in life or concerns that consenting to the vaccine would signal consent to sexual debut or trigger promiscuous behavior. The child’s young age was expressed to influence parents’ perceived risk and need for protection, leading to vaccination delay. Furthermore, the HPV vaccination was reported to be down-prioritized due to other medical/psychological problems, fear of needles, or having been hesitant to—although accepting—other childhood vaccines. Another challenge described was conflicting views within the family, either between parents or between child and parents. School nurses generally felt more confident in addressing minor misconceptions or lack of knowledge. They experienced that further information through dialogue could motivate hesitant parents more effectively than extensive written information. It was also described as important to build a good relationship with the family. However, this could be challenging because of language barriers or lack of time due to part-time employment or a large number of pupils.

*“They [parents] don’t think this is relevant for them, because you only have sex with one and that is who you marry. And that’s hard to get around. I can’t speak against that.”* FG2

#### 3.1.2. Communicating with Outspoken Vaccine-Skeptical Parents

School nurses felt most challenged when communicating with outspoken vaccine-skeptical parents, described as not being interested in further dialogue. Nurses felt less confident in their ability to promote vaccinations due to a more rigid belief system among these parents. Furthermore, past experiences of negative reactions, such as being accused of being intrusive or spreading vaccine propaganda, contributed to nurses spending very little time trying to reach this group.

*“But those who are genuine anti-vaxxers, those who won’t say yes to anything, it’s like… Some families, there’s no use even calling them because they will only get angry at me, that ‘you should stop nagging about that, you know where we stand’.”* FG1

#### 3.1.3. Providing Enough Information but Respecting the Parents’ Own Decision

Participants found that it was challenging to strike a balance between providing hesitant parents with enough information to make a well-informed decision and being respectful of their right to choose as it is a non-compulsory vaccine. They described being worried about either not having done enough or overstepping their mandate. It was also expressed to cause uncertainty that influenced their motivation for future dialogue with vaccine-hesitant parents.

*“It is not often that I actually have talked to parents who have said no [referring to vaccine] in fifth grade, to ask. Because I’m scared that they will feel questioned. I’ve experienced that they became a bit upset when I did. So, it is my way of respecting their decision and then I have several years to offer again and discuss.”* FG4

### 3.2. Interpreting Professional Responsibilities

#### 3.2.1. Defining the Extent of Responsibilities

The school-based vaccination program was regarded as a natural arena for HPV vaccination in terms of accessibility and availability. Participants expressed favorable attitudes towards the vaccine and viewed vaccinations as a part of their professional role as healthcare providers. However, school nurses described uncertainty in terms of the extent of their professional responsibility. Some expressed that this extended to offering the vaccine but that it was the parent’s responsibility to contact the school nurse if in need of more information and, if postponing the vaccination, to request the vaccine at a later time.

*“(…) because vaccinations are really nothing I have to make any assessments about or go in to like, in my profession as much…rather, it’s an offer and I, like, give the pre-written information with a consent form and then you answer questions or refer to the Public Health Agency, full stop.”* FG6

*“If it’s my responsibility to constantly remind and offer, or if you just leave it to the parents to let us know… well I don’t have many who have said no…but I don’t call them up.”* FG6

Others described the importance of active follow-up and of providing as much information as possible, sending additional information such as video links on their own initiative or contacting all hesitant parents for further dialog. They also saw it as their responsibility to offer a new opportunity to vaccinate at a later time if parents had initially declined.

*“Yes, because it feels a little bit like a failure when you don’t get everyone to say yes. Yes, actually because it is within our professional responsibility.”* FG1

Nurses described that knowledge and experience motivate individual initiatives to promote vaccination more actively. They described their professional role as very independent and that they gained knowledge mostly by studying and keeping up to date on their own initiative. Work-related and personal experiences, as well as the situations at the schools, also influenced both the possibility to gain knowledge and the motivation to do so. Receiving more education about HPV and the vaccine from the central school health services was regarded as valuable, especially among those with less work experience.

*“I would very much like to have a lecture about it, actually, maybe at work meetings or a good document that can be used.”* FG7

#### 3.2.2. Lacking Guidelines

A lack of guidelines was noted, which are regarded as a means to ensure the homogeneity of vaccine promotion, support in addressing vaccine hesitancy, and help define the extent of the school nurses’ responsibility. The school health services were compared to child health services or childcare centers, where more extensive guidelines and support materials are given to nurses working with the childhood vaccination program.

Similar work methods were described when informing children and parents: children were informed verbally in class and the focus of content was adapted based on the children’s questions, whereas parents had written information sent home together with the consent form. Participants either used, or were about to start using, digital parental information and consent forms through the schools’ web-based communication platforms. For some, this decreased the workload while others had the opposite experience as it increased the number of forms to collect. By shifting from previously needing just one consent form representing both parents, the digital version from the PHA requires separate forms, one for each parent.

The content of the information given was based on the PHA’s information material. However, participants described using different materials, with the amount of information depending on individual initiatives and available tools. It was seen as important that the information was available in minority languages, and a need for multi-language videos targeting parents who had language or reading difficulties was expressed.

Informing children about HPV was by some viewed as a good opportunity for sexual health education, in general, while others perceived the children not to be mature or old enough. Furthermore, nurses feared that emphasizing the prevention of a sexually transmitted infection could result in negative attitudes towards the vaccine among both parents and children.

*“But for those who decline, then we [state driven schools in the municipality] have just decided that we send out a letter when we go through all vaccinations in eighth grade. Then we send out a new offer to those who declined.”* FG4

#### 3.2.3. Supporting the Child to Be Involved in Decision Making

Children in fifth grade were described as mostly having favorable attitudes towards the vaccine and could recognize the importance of getting vaccinated. School nurses described informing the children in class before sending information to parents, encouraging children to discuss and be part of the decision making together with their parents. If the child wanted to get vaccinated and parents disagreed, school nurses opined that, in general, fifth-graders were too young to decide for themselves without parental consent. However, as the child became older, the dilemma of considering the child’s wishes was experienced as more challenging. School nurses described various regional age limits—12, 13, 15, and 16 years—for when children could decide about vaccination without parental consent, while other regions had no stated limit. An uncertainty about practice and regulations for the evaluation of the child’s maturity level was expressed. The quote below illustrates a situation when the child had wanted to get vaccinated when she was first offered, but she was not allowed to decide for herself until the age of 16 according to regional guidelines.

*“I thought that in ninth grade she was mature enough to make the decision. I believed that, that when she was in ninth grade, she was fully capable to make that decision, but I was not allowed [according to supervisor] to vaccinate.”* FG4

### 3.3. Creating a Safe Space for the Individual Child

#### 3.3.1. Using Strategies to Overcome Children’s Fear of Needles

That children were well informed about HPV and the vaccine as well as the practical aspects of the vaccination was seen as vital, both for the child’s right to be involved and in order for the individual child to feel comfortable and safe during vaccination. School nurses perceived that fifth-graders also are at a sensitive age and that the vaccination is fairly painful. Participants described using different strategies to prevent children’s fear of needles from becoming a barrier to vaccination. These strategies included practical preparations and adaptions to meet individual needs, for example: inviting the child to talk in private, practicing the procedure, offering the option of bringing parents or a close friend, or receiving the vaccination at a different time than the rest of the class to avoid peer pressure. Individually adapted strategies were described as time consuming but very important as the vaccination needs to be given twice and fear of needles is common among children. Regardless of this, the parent’s motivation to vaccinate was crucial when trying to overcome the child’s fear. School nurses were also able to refer children to child health clinics for play therapy, but it was experienced that some clinics did not have time for this. Another challenge was improving collaboration with other healthcare services for children with other medical needs, with the aim to coordinate the vaccination with the child’s other medical procedures.

*“...At the hospital it is not prioritized… So, because of that I have girls who have not taken the second dose HPV, and actually one boy who has not taken dose two I think… doesn’t dare. I think it’s so sad…”* FG7

#### 3.3.2. Being Confronted with Gender Inequities of the Pan-Gender Vaccination Program

School nurses described that the pan-gender vaccination program is more inclusive and its introduction a longed-for reform. They expressed that it was important that the HPV vaccination was offered equally, and that it was a collective responsibility to prevent HPV-related disease and a right to be protected regardless of gender and sexual preferences. It was also seen as being for all, including children with non-majority gender identification. They expressed that the acceptance among boys was surprisingly good and that the reform had increased vaccine acceptance among both children and parents in general. The fact that older boys not included in the introduction of the pan-gender vaccination program are not offered catch-up vaccinations free of charge through the school-based vaccination program was described as unfair, and school nurses had received complaints from older boys and their parents. Some perceived that boys included in the pan-gender vaccination program would not get a second opportunity to be vaccinated if initially declining in fifth grade. They also had experienced angry reactions from parents believing it was the school nurse’s decision rather than a decision by health authorities.

*“I must say that when the boys got it, when it was implemented for them in the childhood vaccination program, I received unbelievably many calls from parents with older children who wished to vaccinate their child, they thought it was very unfair.”* FG4

#### 3.3.3. Supporting Unvaccinated Children

Another challenge was supporting children who do not get vaccinated. This support included tackling peer pressure, handling discretion about vaccination status, informing the child about the possibility to decide for themselves when they are older, and facilitating discussion between the child and parent. The latter was described to be difficult since it could be regarded as intrusive in the eyes of the parents, especially if the child wanted to be vaccinated but the parents decided against it. Experiences handling worry among peers about the consequences of their friends not being vaccinated were also described. Furthermore, more extreme examples were described such as when vaccine-skeptical parents pressured the school nurse and school management to exclude the child from receiving information about the vaccine.

*“I think it’s difficult when parents have said no but then the children come and ask ‘can’t you vaccinate me anyway? Mom and dad don’t have to know or find out.’... then I handled it by letting the pupils call their parents from my phone and… talk to their parents.”* FG7

## 4. Discussion

### 4.1. Main Findings

Our results show that school nurses encountered various reasons for parental vaccine hesitancy and perceived the greatest challenges when addressing either skepticism of vaccines in general or moral and religious reasons for hesitancy. Difficulties in the promotion of the HPV vaccine were influenced by uncertainty about the extent of professional mandates and responsibilities. Furthermore, school nurses expressed challenges to protect the individual child’s wishes regarding vaccination while maintaining good relations with the entire family. The pan-gender vaccination program was viewed to be successful and increased HPV vaccine acceptance in general, while inequities remain as older boys are not offered catch-up vaccination.

### 4.2. Strengths and Limitations of the Study

The criteria for assessing the quality and trustworthiness of the conducted study were considered, i.e., credibility, dependability, conformability, and transferability [34,35]. Due to the pandemic, most interviews were conducted digitally, whereby distractions of surroundings were more difficult to control. However, digital meetings were standard at the time and participants stated that they felt comfortable with the format. For each FG, 5–9 school nurses were recruited. Due to substantial sick leave during the COVID-19 pandemic, and in some cases heavy workload, drop-outs resulted in groups of 3–7 participants. Smaller groups may decrease the desired interaction of FGs, but on the other hand, suit digital data collection better [36]. Rich data were generated from all interviews and the overall content was similarly described. According to Malterud (2016), information power can be evaluated by the richness of data and highly specific experiences of the phenomenon among participants [33]. All participants had experiences of meeting vaccine-hesitant parents and general vaccine skepticism, and after seven FGs, it was considered that information power was reached. Efforts to include municipalities and schools with lower HPV vaccine coverage were made by using official statistics available at the time. At the school level, this was more difficult to control beforehand. It can be speculated that nurses working in schools with more problems and school nurses with less favorable attitudes toward the vaccine may have declined to participate. As in all qualitative research, the aim is not to generalize. However, the representative sample of nurses working in socio-demographically diverse areas, in private as well as public schools, and the nurses’ varying work experiences strengthen the transferability of the results.

### 4.3. Findings in Relation to Other Studies

A large number of studies have previously reported the complex variety of parental concerns and barriers to vaccine acceptance [9,15,16,37,38,39]. The recommendations of HCPs are important for vaccine acceptance and in school-based HPV vaccination program school nurses are uniquely situated to advocate for the vaccine both to children and their parents [23,40,41].

Our results confirm that school nurses have an independent role with freedom to tailor context-specific solutions, but also, to a large extent, have the responsibility to gain knowledge and keep up to date themselves [23,27]. HCPs’ own knowledge and attitudes have been shown to be important for how vaccines are promoted. Furthermore, having the capacity and means to educate about the virus, related diseases, and vaccination is crucial for HCPs’ effective vaccine promotion [21,24,27,42].

In the present study, vaccination acceptance was generally perceived as good with only a few parents declining in each school class. This may reflect higher vaccination coverage among fifth graders in the schools represented by participants. However, experiences of increased vaccine acceptance over time may have influenced school nurses’ attitudes about the success of the vaccination program, even though vaccination goals had not yet been reached. It is important to take into consideration that the number of fifth-graders in different schools varied, some having several parallel classes, and others only one. Consequently, “a few” in a large school affect coverage rates to a lesser extent compared to a smaller school with fewer fifth-grade students.

Participants felt that they were less subjected to parental concerns compared to when the vaccine was first implemented. They also felt confident in their competence to answer general questions about the vaccine. This is encouraging since school nurses previously have expressed the need for more knowledge about HPV and the vaccine [18,27]. Still, the informants experienced challenges when confronted with more specific concerns or moral reasons for declining the vaccine. Underlying concerns regarding the vaccine’s protection against an STI have previously been reported and seem to remain a barrier, causing an ambiguity towards emphasizing sexual transmission of the virus when informing parents and children [15,18,43]. Moral concerns seem to be important predictors of vaccine hesitancy and should be taken into consideration when communicating with hesitant parents [44]. Therefore, increased support to strengthen confidence in provider-parent vaccine discussions may be of more value. However, less experienced HCPs may benefit from a combination of support and educational interventions, which have been shown to be effective in previous reviews [25,43].

Previous studies from the US found that HCPs tend to offer the HPV vaccine as an optional or additional vaccine that may be delayed, instead of strongly recommending it. Furthermore, HCPs avoided communicating with hesitant parents [43]. The present study found similar results; i.e., some school nurses did not actively reach out to hesitant parents and felt confident that they had ample time to offer catch-up vaccinations. This is worrying since the vaccine, for the best level of protection, should be distributed before exposure to the virus, i.e., before sexual debut [6]. When HCPs perceive their role as opinion leaders who can have an impact on vaccination rates and public health, they tend to have more favorable attitudes to the vaccine and to provide information to parents [45,46]. In a recently published study, Swedish school nurses described the dilemma of being proactive while remaining neutral [47]. Similarly, as in the present study, this seems to reflect uncertainty about the professional role. We believe that the HCPs’ role should *not* be neutral: their information should be transparent and evidence-based, resulting in their professional recommendation even though they are required to be respectful of individuals’ autonomous decision making. Furthermore, as reported in a recent Swedish study, nurses expressed a need for standardized work methods, so that vaccine promotion would not be dependent on individual school nurses’ initiatives [47]. In the present study, child health care services were perceived to have better support and guidelines to address vaccine hesitancy, as compared to school health services. A practical example concerned separate consent forms for both parents, which are not needed in that setting.

Vaccine acceptance was mainly described as a consequence of the general population’s high trust in the national vaccination program (NVP) and the time that has passed since implementation. Furthermore, the pan-gender program was perceived to have had a positive effect on vaccine acceptance in general. These findings corroborate previous studies showing that parents’ trust in the safety of the HPV vaccine would be higher if it was offered to both boys and girls [48,49]. On the other hand, experiences of parents declining due to a lack of knowledge of the inclusion of HPV in the NVP were expressed. Furthermore, written information and consent forms were often formulated with a focus on the vaccination as an “offer”, creating this misconception. Therefore, the HPV vaccination’s inclusion in the NVP should be emphasized in information aimed at parents.

With increased vaccine acceptance it is of great importance that HCPs are knowledgeable and confident in their ability to meet parental concerns and misconceptions when they arise. That school nurses recognize their key role is important for the resilience of NVPs since hesitancy can quickly become problematic due to the spread of misinformation, and political and societal events as seen in Denmark and Japan where vaccination rates, in highly functioning HPV programs, dramatically decreased [50,51]. Previous studies of educational interventions aimed to increase parental knowledge and favorable attitudes have shown that dialogue and face-to-face interventions may be more successful than written information [52]. This was also expressed by nurses in this study who worked actively with follow-up dialogue with hesitant parents. According to the UN Convention on the Rights of the Child, which is elevated to Swedish law, children have the right to equal quality of care, to privacy, and to personal integrity, as well as the right to be heard [53]. The present study found extreme examples such as when vaccine-skeptical parents pressured the school nurse and school management to exclude the child from vaccine information, thereby putting the school nurse in a vulnerable position. School nurses have previously expressed the importance of having close cooperation with principals and teachers in order to be successful in their health-promoting task [54].

Children have a lawful right to be involved in decisions regarding their own health, and their wishes should be taken into consideration according to their degree of maturity. Swedish law does not define any specific age when this maturity is achieved. Consequently, in cases when parents opposed vaccination despite their child’s wishes, school nurses lacked confidence in allowing the child’s wish to be final. This is in line with previous studies from Sweden and the UK [55,56,57]. Furthermore, in some cases, school nurses felt restricted due to regionally applied age limits, indicating that such strategies are problematic.

## 5. Conclusions

Altogether, these findings suggest a need for increased support for school nurses in their role as HPV vaccine promotors, including homogeneity in work methods and clarification of regulations for children’s participation in decision making. School nurses play a key role in the HPV vaccination process and should feel supported and confident in their responsibilities. HCPs with less experience may particularly benefit from training and guidance materials on how to address vaccine hesitancy. It may very well be the case that hardcore overall vaccine-skeptic families are impossible to reach, but with better guidelines and training, the larger group of hesitant parents may still be motivated. This way, herd immunity may be reached and HPV-related cancer eliminated.

## Figures and Tables

**Table 1:** Example of data analysis.

Interview Transcript	Initial Coding	Sub-Category	Category
I have offered to actively contact them [parents] again to see how things are going. Because they are busy and then they should remember this also. So, I choose to actively get in touch with them again, with another offer (FG 2)	Choose to offer again actively	Defining extent of responsibilities	Interpreting professional responsibilities
they are welcome to contact me if they change their mind, it is up to the parents to get in contact (FG 2)	It is up to the parents		

**Table 2:** Participants’ characteristics (*n* = 35).

**Age (Years)**	
30–49	13
≥50	22
**Country of Birth**	
Sweden	33
Other	2
**Education**	
Registered nurse	2
Specialist degree in Primary health care	22
Specialist degree in Pediatric nursing	10
Other specialist degrees in nursing	1
**Experience of HPV Vaccination (Years)**	
≤2	10
3–9	14
≥10	11
**Work Experience as School Nurse**	
≤5	16
≤10	10
>10	9
School Management of Represented Schools *	
Private	2
State	45
**Range of Total Number of Students in Represented Schools**	200–708

* 10 participants were employed at ≥2 schools.

**Table 3:** Results of data analysis.

Categories	Addressing Various Reasons for Vaccine Hesitancy	Interpreting Professional Responsibilities	Creating a Safe Space for the Individual Child
Sub-categories	Encountering hesitant parents	Defining the extent of responsibility	Supporting unvaccinated children
	Communicating with outspoken vaccine-skeptics	Lacking guidelines	Using strategies to overcome fear of needles
	Providing enough information but respecting parents’ own decision	Supporting the child to be involved in decision making	Being confronted with gender inequities in the pan-gender vaccination program

## Data Availability

The data presented in this study are available on request from the corresponding author. The data are not publicly available due to ethical reasons and privacy of participants.
